# The Most Common Environmental Risk Factors for Equine Asthma—A Narrative Review

**DOI:** 10.3390/ani14142062

**Published:** 2024-07-14

**Authors:** Anna Mańkowska, Dorota Witkowska

**Affiliations:** Department of Animal Welfare and Research, Faculty of Animal Bioengineering, University of Warmia and Mazury, 10-719 Olsztyn, Poland; dorota.witkowska@uwm.edu.pl

**Keywords:** equine asthma, environmental factors, welfare, horses, respiratory tract

## Abstract

**Simple Summary:**

Equine asthma is a major disease affecting the health and welfare of horses worldwide. There are many factors in the stable environment that directly lead to the exacerbation of asthma symptoms and indirectly affect the well-being of horses. We conducted a thorough analysis of scientific articles published over the last 20 years on potential environmental factors influencing the development and course of equine asthma. In conclusion, numerous environmental factors have a significant impact on the well-being of horses, and their control helps prevent the occurrence of asthma in horses or alleviate its symptoms. We believe that the information collected in our review will help improve the welfare of horses.

**Abstract:**

Equine asthma is a popular subject of research. Many factors influencing the methods used to improve the welfare of asthmatic horses remain unclear. This study reviews scientific articles published after 2000 to collect the most important information on the terminology, symptoms, and potential environmental factors influencing the development and course of equine asthma. Our work highlights the impact of environmental factors on the severity of equine asthma and why these factors should be controlled to improve treatment outcomes. The present article provides horse owners and veterinarians with valuable information on how to improve the well-being of horses that are at risk of developing asthma symptoms.

## 1. Introduction

Asthma, also referred to as a chronic inflammation of the respiratory tract, is a ubiquitous health problem in humans and animals [1,2,3]. Regardless of their use, horses are often affected by asthma symptoms with variable severity [4,5]. Considerable advances have been made in asthma research over the years, but many factors affecting the treatment of asthma remain unexplained [6,7,8,9]. Some asthmatic horses do not respond positively to treatment, and the severity of clinical signs often leads to premature retirement or even euthanasia [10]. 

Despite constant attempts to determine the cause of equine asthma, it is still not clear why the disease affects some horses and not others. Strong genetic predispositions have been noted in some families. Borowska et al. [11] analyzed the hereditary component of equine asthma. Maternal and paternal lines were correlated with a predisposition to equine asthma. According to Leclere et al. [12], breed has no influence on the occurrence of chronic respiratory disease. In contrast, Couëtil et al. [13] analyzed 1444 patient cases and concluded that breed was a predisposing risk factor. They suggested that Thoroughbred horses are more susceptible to asthma than other horse breeds. 

Gerber et al. [14] identified a gene involved in the development of equine asthma. The results suggest that the main gene and its location on chromosomes may differ between families: the mode of inheritance was autosomal recessive in one of the studied families, and it was autosomal dominant in other families. The mechanism of equine asthma is not fully understood, but it is well known that environmental factors play the most important role in alleviating the symptoms of this disease, and management of the equine environment directly contributes to changing the severity of equine asthma and actually improving their health and well-being. The current review of the literature on equine asthma will support the identification of environmental factors that influence the development and course of equine asthma.

To review the literature and research findings on both mild/moderate and severe equine asthma, we focused on studies that have been published in the last twenty years. The literature review was divided into paragraphs containing basic information about equine asthma as a disease, including terminology, disease symptoms, diagnostic methods, and potential environmental factors influencing the development and course of the disease. In addition, the conclusions from the reviewed studies describing the impact of various environmental factors on the welfare of horses suffering from or potentially at risk of asthma are presented in tables. We hope that our work will contribute to a better understanding of equine asthma and will enable horse owners to minimize the impact of environmental factors that cause the disease.

## 2. Materials and Methods

PubMed and Web of Science databases were searched in March and April 2024 for scientific articles. The following search terms were used: “equine asthma”, “chronic obstructive pulmonary disease”, “recurrent airway obstruction”, “inflammatory airway disease”, “small airway disease”, and “heaves”. Individual articles were divided into four subcategories: (a) nomenclature, (b) diagnostic methods, (c) selected environmental factors, and (d) potential predisposing factors other than environmental factors. The literature review focused on environmental factors. For this reason, the analyzed databases were searched using the above terms in combination with the following keywords: “temperature”, “humidity”, “season”, “air pollutants”, “feeding”, “bedding”, and “microbiological contamination” to identify additional articles. The above phrases were also entered into the Google Scholar database to find additional sources. The results were filtered manually and used to improve the quality of the review.

All citations identified in the search were imported into the EndNote Online citation manager (Clarivate Analytics, London, UK). Duplicate citations were removed manually. Subsequently, a two-stage screening process was performed to assess the relevance of the sources identified in the initial search. The title and the abstract were screened for relevance in the first step. In the second step, the selected articles were reviewed in full.

To extract the key information from selected articles, the results were collated and summarized in terms of the different subcategories of the presented topic. The extracted information was used to present the results in a narrative form.

Combining both databases, the search strategy yielded 369 records in total, including both peer-reviewed articles and doctoral dissertations. After the removal of duplicates, 271 records were screened by title and abstract. Moreover, 208 full-text records were then assessed for eligibility, and 144 of those were included in the final narrative review. Most articles were rejected because they discussed the diagnosis and treatment of equine asthma, which were outside the scope of this review.

## 3. Description of Equine Asthma

### 3.1. Nomenclature of Equine Asthma and Its Symptoms

Equine asthma is a chronic disease of the respiratory tract. Over the years, various terms have been used to describe asthma in horses. A simplified terminology limited to the three basic types of asthma has recently been introduced. At present, equine asthma is classified as mild/moderate or severe, depending on the severity of the symptoms [6,10]. Chronic inflammation of the lower respiratory tract is generally divided into mild to moderate asthma (mild/moderate asthma; MEA) and severe equine asthma (SEA), depending on the degree of advancement and the presence (or absence) of selected symptoms. Mild/moderate asthma is associated with the obstruction and inflammation of the lower respiratory tract [15], and it affects 80% of the global horse population [4]. In mild/moderate equine asthma, there are no characteristic clinical signs of pulmonary disease, but it most commonly presents as exercise intolerance [15]. Severe asthma is irreversible because it leads to the permanent reconstruction of the respiratory tract [16]. Horses suffering from severe asthma develop chronic cough even when at rest [17]. However, sick animals may experience periods of clinical remission, followed by a phase of exacerbation lasting months or years [16].

In the past, equine asthma was also referred to as heaves, recurrent airway obstruction (RAO), equine chronic obstructive pulmonary disease (COPD), inflammatory airway disease (IAD), including tracheal and bronchial IAD, small airway disease, or chronic bronchitis [6,10]. Due to considerable variations in the nomenclature and the lack of cohesive indications for classifying differences between individual diseases, such as differences in the pathogenesis or severity of symptoms, these terms were replaced with three terms that distinguish the stages of disease severity well [6]. Equine asthma was also further divided into the following severity classes: mild/moderate and severe. 

To account for the season, in case of severe asthma in horses, additional terms were introduced, such as “summer pasture-associated obstructive pulmonary disease” (SPAOPD) and “hay/barndust asthma”. Pollen has been implicated as a trigger for clinical manifestations of SPAOPD [18]. A higher temperature in spring and summer is associated with increased concentrations of airborne pollens and molds which can exacerbate the symptoms of SPAOPD [18,19]. In the case of summer pasture asthma, horses show symptoms of severe asthma despite being outdoors [20,21]. They are probably also allergic to dust, mites, or other allergens, which are triggering factors [22]. The consequence of this is the need to keep horses with SPAOPD indoors during the summer [20]. Another phenotype of severe asthma is hay/barn dust asthma. In horses suffering from this SEA phenotype, the exacerbation of symptoms, unlike in pasture asthma, occurs when horses stay in stable buildings, where they are exposed to dust mainly from bedding and hay or other roughage [23]. These horses stay outdoors in favorable weather conditions, especially in the summer, to minimize the risk of dust exposure [24]. In addition, changes in the nutrition system may prove helpful in eliminating triggering factors.

The most characteristic symptoms of equine asthma include coughing, mucous nasal discharge, shortness of breath, wheezing, and short inhalation–exhalation [25]. The increased work of respiratory muscles, which can be seen in the movements of the horse’s sides, is also usually emphasized both during exercise and at rest. Excess mucus in the equine respiratory tract increases resistance to air flow and leads to breathing problems [6]. In a study by Gehlen et al. [26], the occurrence of RAO in horses was associated with histopathological changes in their skeletal muscles. Pathological changes in skeletal muscles (fiber atrophy or fiber hypertrophy, myofibrillar degeneration, hyperplasia of mitochondria, and ragged-red-like fibers) were identified in most horses with RAO. Muscle changes associated with respiratory diseases are also supported by the research of Herszberg et al. [27], who reported that the mass of airway smooth muscles was nearly three times higher in heaves-affected horses. 

Despite the fact that the nomenclature of equine asthma has been simplified, the severity of the disease is difficult to classify in veterinary practice because clinical symptoms are non-specific, the existing diagnostic techniques are not sufficiently sensitive for assessing lung function, and portable equipment for field work is not available [15].

The term “moderate equine asthma” refers to cases with clinical symptoms such as coughing or lung sounds. Severe equine asthma is a chronic respiratory disease that affects up to 20% of horses older than 7 years [28]. Disease symptoms are triggered by high concentrations of airborne allergens [17,29] to which horses are constantly exposed in the stable environment. Dust containing fungi, molds, and particles of allergenic plants are ubiquitous in places where horses are kept. The most common symptoms of equine asthma include coughing, shortness of breath, wheezing, short inhalation–exhalation, and increased frequency of respiratory muscle contractions [6,30]. In asthmatic horses, increased respiratory effort is observed at rest [31]. Horses showing the above symptoms have a reduced exercise capacity, which compromises their sports performance [4,32]. Equine asthma may also involve bronchospasms, the thickening of the airway wall, markedly increased mucus secretion, and the physical remodeling of the airways [30,31]. Histopathological changes in the bronchi are observed in approximately 90% of racing horses with asthma [32]. Asthma undoubtedly hinders the free circulation of air in the lungs, causes hypoxia, and undermines the exercise capacity of horses [33,34]. Horses with severe asthma also experience breathing problems at rest, which decreases their quality of life and overall well-being [5,35]. Clinical symptoms vary across seasons and housing systems, and symptoms tend to exacerbate in winter when horses spend more time in stables [9].

### 3.2. Methods of Diagnosing Equine Asthma

Equine asthma is diagnosed based on the animal’s medical history, the results of a standard clinical examination, the presence of clinical symptoms indicative of the inflammation of the lower respiratory tract, and the results of bronchoalveolar lavage fluid (BALF) cytology [26] or the tracheal wash (TW) procedure [6,31,36,37]. The preferred diagnostic methods involve assessments of clinical symptoms and BALF cytology [31]. Lower respiratory tract obstructions pose a diagnostic challenge in veterinary practice [38,39]. Current techniques are not sensitive enough and often cannot be applied in the field [6,40], which is why patients have to be transported to a veterinary clinic for diagnosis [41,42]. The severity of equine asthma can also be assessed with the use of various supportive and supplementary diagnostic methods, including respiratory endoscopy [43], endobronchial ultrasonography [31], endobronchial biopsy [44], computed tomography [45], pulmonary function tests [45], or arterial blood gasometry [46,47]. Some authors have relied on clinical staging to facilitate the assessment of the severity of equine asthma. Clinical staging is a system for assessing disease severity based on the results of various respiratory tests [47,48]. In this approach, disease severity is rated based on the results of comprehensive respiratory tests using various techniques. In a study by Simões et al. [47], the observed variables were classified and allocated to one of five stages: stage 0 (horses without respiratory disease), stage 1 (horses with the least-severe form of RAO), stage 2 (a mild presentation of RAO), stage 3 (a moderate presentation of RAO), and stage 4 (horses with the most severe form of RAO). Thus, disease staging provides the owners of asthmatic horses with an objective tool for evaluating the animals’ respiratory health, and it is also helpful in increasing the awareness of the relevance and effectiveness of environmental management in maintaining remission.

Laumen et al. [49] proposed the Horse Owner Assessed Respiratory Signs Index (HOARSI), which can be used by non-veterinarian horse caretakers as a complementary method in clinical diagnosis. The HOARSI questionnaire is usually used at the beginning of research to quickly divide horses into groups with different severities of asthma [50,51], and when combined with an evaluation of housing and environmental conditions, it may contribute to improvements in the management of asthmatic horses. The HOARSI is based on an owner-reported clinical history and has been used to investigate RAO genetics in a large sample. The owners complete a standardized questionnaire including questions about gender, age, feed, bedding, time spent outdoors, performance, and signs of respiratory disease (coughing, nasal discharge, type of breathing, and performance) [49]. Horses are graded on a scale of one to four, in which grade 1 denotes historically healthy horses, and grade 4 represents severely affected individuals [29,49]. Owner reports of respiratory signs have shown that the HOARSI test has a good level of repeatability. The rate of disagreement between the original and repeated owner interviews is less than 1% [49]. The HOARSI questionnaire has been successfully used in research to distinguish between mild/moderate and severe asthma [50,52,53].

### 3.3. Selected Environmental Factors That Contribute to Equine Asthma

#### 3.3.1. Temperature and Air Humidity

Thermal and humidity conditions, balancing the amount of evaporated water and heat released from the equine respiratory tract, influence the processes occurring in the trachea and pulmonary bronchi. The ranges of air temperature and relative humidity that are well tolerated by horses are from below 0 °C to above 20 °C and from 60% to 80% humidity [54]. Pasture-associated severe equine asthma is an environmentally induced respiratory disease observed during the warm season, characterized by reversible airway obstruction, persistent and nonspecific airway hyperresponsiveness, and chronic neutrophilic airway inflammation [55,56]. Symptoms may become more severe when horses are grazing due to their exposure to dusty allergenic plants. An increase in environmental temperature and humidity (determinants of humid air enthalpy that are strongly associated with pollen and spore concentrations in air) negatively affects lung function in asthmatic horses during disease flare-ups and further worsen airway obstruction [19]. During a flare-up, the breathing frequency increases in asthmatic horses compared with that of healthy animals, and a considerable part of the tracheal mucosa is often covered with mucus, which could hamper thermoregulation in horses. The clinical condition of horses often deteriorates with an increase in ambient temperature.

Bullone et al. [19] reported a correlation between high temperature and the severity of clinical signs in severe equine asthma. Airway obstruction worsens when increased temperature and/or relative humidity levels prevent heat dissipation, which could suggest that a heat-induced bronchospasm is implicated in the pathobiology of EA. The authors of [19] provided evidence supporting the correlation between daily outdoor temperature, relative humidity, and airborne pollen vs. spore levels. Airborne pollen, but not at significant concentrations, is correlated with clinical scores, which suggests that it could play a role in disease severity. Interestingly, a significant correlation was reported between clinical scores and pollen concentration on the previous day, as the horses spent the afternoon outside and were assessed early in the morning. On average, the outdoor pollen concentration was three times higher on a hot day than on a warm day when pulmonary function tests were performed [19]. The results of BALF analyses vary across seasons; relative mast cell counts increase in spring, whereas eosinophilic and neutrophilic responses are more common in summer [57]. In turn, Davis [58] found that exercise in cold weather can lead to asthma-like airway diseases in horses. Another study revealed that the winter season may contribute to the exacerbation of asthma symptoms in horses, which may be caused by feeding horses outdoors with hay from round bales [9]. Even mild winter weather conditions with lower temperatures, fewer minutes of sunlight, and a higher humidity were associated with significantly higher BALF neutrophil counts and significantly higher mucus scores in stabled clinically healthy horses [59]. However, Araneda and Cavada [41] did not find any associations between air humidity and respiratory performance; it should be emphasized that regardless of the type of construction, the stable is a closed facility with limited space, but in harsh climates, it is “a necessary evil” [54].

The influence of climatic factors on the well-being of asthmatic horses is presented in Table 1.

#### 3.3.2. Air Pollutants

The risk of disease exacerbation increases during the winter months [28] when horses are stabled for extended periods and exposed to endotoxins, molds, mites, and other types of particulate matter in hay and straw [60]. Horses that spend more than 15 h/day outdoors during winter are at higher risk of asthma exacerbation [28]. However, the worsening of clinical symptoms has also been reported during the summer months [61], even when horses were kept mostly outdoors. Similar results were reported by Bullone et al. [19].

During exercise and at rest, horses are exposed to many potentially harmful substances in the air, including organic and inorganic particulate matter (<PM10, especially <PM2.5); gaseous admixtures such as ammonia (NH_3_), nitrogen oxides (NO_2_ and NO), sulfur dioxide (SO_2_), carbon monoxide (CO), and ozone (O_3_); and microorganisms (bacteria and fungi) as well as their toxins and viruses. These components are inhaled by horses and have a negative impact on their health, due to the sensitivity of the respiratory tract [54,62,63,64,65]. Some pollutants have been found to affect lung function [62,66,67]. It should be emphasized that inhaled air also contains naturally occurring pollutants. Their concentrations vary depending on the time of day, season, and geographical location [63]. Araneda and Cavada [62] studied the influence of environmental factors, including pollutant concentration, humidity, and temperature, on the performance of racing horses. They found that good performance in sports was negatively correlated with the concentration of pollutants in the air and confirmed that air pollutants compromised the performance of racing horses. 

##### Harmful Gaseous Admixtures in Air

Kwiatkowska-Stenzel et al. [65] found that stable air contained gases that can be dangerous to horses, including ammonia (NH_3_), nitrogen dioxide (NO_2_), sulfur dioxide (SO_2_), carbonyl sulfide (COS), hydrogen cyanide (HCN), methane (CH_4_), and carbon monoxide (CO). The concentrations of harmful gases in stable air fluctuated throughout the day. Depending on their source, the highest concentrations of naturally occurring gaseous compounds were noted in the early morning (4.00 a.m.), whereas anthropogenic emissions peaked at noon.

1. The main sources of harmful gaseous admixtures in air

Ammonia (NH_3_) and carbon dioxide (CO_2_) are frequently encountered in animal buildings [68]. Manure, a combination of feces and urine, produced in large amounts by horses is the source of harmful gases, such as ammonia [69]. Carbon dioxide is a natural component of air and occurs at a concentration of 300 ppm. Carbon dioxide is produced in the process of respiration, and therefore, with a large number of animals in the building, the concentration of carbon dioxide is much higher than in atmospheric air [68].

2. The impact of gaseous admixtures on horse health

Researchers emphasize that many gases that are harmful to both animals and humans are odorless [65]. Oxides (NO and NO_2_) induce a pro-inflammatory response and increase airway resistance [70]. In turn, CO affects performance by inducing changes in the conjugation of oxygen to hemoglobin [71], which may affect the functioning of the heart and other organs [72]. SO_2_ is also a pro-inflammatory, irritating substance that promotes bronchoconstriction [73]. Ammonia (NH_3_) is odorous and one of the most common toxic volatile compounds in stables. It has a direct effect on the respiratory system and is one of the main factors leading to the occurrence of respiratory disorders. NH_3_ affects the mucous membranes of the mouth, eyes, and respiratory tract [74]. Ammonium hydroxide formed on moist surfaces can cause the inflammation of the respiratory tract membranes, decrease local immunity, and increase infection rates [54,65]. Therefore, the concentration of ammonia in the stable air should not exceed 10 ppm [75,76,77]. At higher concentrations, CO_2_ weakens the respiratory defense mechanisms and increases susceptibility to respiratory diseases. CO_2_ is considered to pose a serious threat to health and life at concentrations higher than 10,000 ppm [68].

3. Methods of gaseous admixture mitigation

Carbon dioxide levels in buildings are a robust indicator of ventilation efficiency [68]. Guidelines for horse stables suggest four to eight air changes per hour to reduce ammonia. With the inevitable seasonal arrival of sub-zero temperatures, stable buildings are often tightly closed and sealed to prevent failures related to water freezing in the water supply. Then, the lack of ventilation contributes to excessive concentrations of harmful gases in the stable. Research by Elfman et al. [78] on the efficiency of mechanical ventilation in a stable showed that the carbon dioxide content of the stable air was reduced by half. It is important to control temperature and humidity because NH_3_ emissions increase with increasing values of these parameters. It is advisable to remove the manure when the horses are out of the stable because activities such as mucking out and sweeping the stable lead to increased concentrations of ammonia and airborne particles [64,77,78,79]. In addition to ensuring adequate ventilation and regular manure removal [80], the type of bedding material also influences the levels of harmful gases in the stable. The use of shavings as bedding decreases NH_3_ concentrations, compared with straw. Test results indicate that peat used as a bedding material binds to ammonia very effectively, helping to limit its release into the air [81]. An effective solution is to use an acidifying additive (with a pH below seven). Acidifiers change the activity of microorganisms by reducing NH_3_, facilitating the conversion of NH_3_ to non-volatile ammonium (NH_4_). Another solution may be the use of zeolite—a natural mineral that binds NH_3_. Zeolite is non-toxic, non-corrosive, moisture-absorbing, and easy to apply. Selected NH_3_-degrading microorganisms may also help reduce the amount of NH_3_ present in feces and the environment. Similarly to zeolite, beneficial microbes are also natural, safe, and non-corrosive. It should be noted that a high dietary protein intake increases the ammonia content of manure. Therefore, if possible, limiting the protein supply will help reduce the amount of ammonia in manure. In addition, the type of flooring affects the absorption of excreta. Wooden or clay floors without an appropriate barrier may become saturated with urine. The use of seamless or interlocking stall mats can minimize this problem [80].

##### Microbial Air Contamination

Microbial air pollution plays an important role in assessments of animal hygiene and as a consequence, affects the well-being of horses [82]. However, legal regulations do not specify the permissible levels of microbial contamination in farm buildings. There are scientific sources that suggest threshold limit values (TLVs) for bioaerosol concentrations in employee facilities of 5.0 log_10_ cfu/m^3^ for bacteria and 4.7 log_10_ cfu/m^3^ for fungi [68]. Dutkiewicz et al. [83] found that animals are more susceptible to disease when the concentration of airborne microorganisms exceeds 3.0 log_10_ cfu/m^3^. Despite the fact that microbial air pollution markedly affects the respiratory system of horses and influences their well-being, microbial concentrations in stable air have not been researched sufficiently to date [78,82,84]. 

Indoor temperature, humidity, and season affect the concentrations of different bacterial and fungal species in the air [82]. Witkowska et al. [82] found that the concentrations of air-borne microorganisms peaked in summer when the temperature and humidity in the stable were high. In the cited study, the average counts of outdoor bacteria also were highest in summer and lowest in spring. However, microbial concentrations in indoor air can also be high in winter when stables are closed on cold days to minimize heat loss [78].

1. The main sources of microbial contamination of air

The most common sources of microorganisms in a stable are the horse manure, bedding material, feed, and horses’ body surfaces; in addition, horses actively expel them, e.g., when sneezing or coughing. Microorganisms enter the stable through the ventilation air as a result of gusts of wind that carry them away from various environments (soil, water, waste, or plant and animal surfaces). Most of them are saprophytic flora (cocci, lactic acid bacteria, molds, etc.) [68,82]. *Penicillium* spp., *Aspergillus flavus*, *Aspergillus fumigatus*, yeasts, and *Fusarium* spp. [82] are the most ubiquitous fungi in stables [82,83,84,85]. It should be noted that *Penicillium* spp. and *Fusarium* spp. produce toxins that are dangerous to animal health [82]. Mostafa et al. [86] identified *Eurotium*, *Wallemia*, and *Cladosporium* molds in the stable environment and concluded that the latter two genera could originate from straw and hay. Lenart-Boroń et al. [87] identified *Wallemia sebi*, *Aspergillus penicillioides*, and *Epicoccum nigrum* fungi in stable bioaerosols.

2. The impact of microbial contamination on the equine respiratory system

The above fungal species are highly allergenic, and they may be involved in the pathogenesis of equine RAO. According to Dauvillier et al. [88], fungi present in the air in the stable environment have a negative effect on the development and aggravation of symptoms of respiratory inflammation in horses. A positive fungal culture was obtained in more than half of the horses, and these had a two-fold higher risk of developing IAD than horses without positive fungal cultures. An equally significant increase in the risk of the exacerbation of equine asthma occurs when a horse is exposed to viruses that cause respiratory diseases. A study by Houtsma et al. [89] confirmed the association between viral respiratory infection and the development or exacerbation of IAD. A relationship was observed between the occurrence of rhinoviruses A and B and herpesviruses and the occurrence of IAD.

Bacteria and fungi are commonly present in the respiratory system of horses, and the species composition and concentrations of these microorganisms differ in various segments of the respiratory tract [90]. The presence of *Streptococcus zooepidemicus*, *Streptococcus pneumoniae*, *Actinobacillus* spp., and *Mycoplasma equirhinis* bacteria in tracheal washes was associated with mild equine asthma, which suggests that the composition of the respiratory microbiome may be implicated in the etiology of the disease [91]. On the other hand, in a study by Mete and Özgür [92], although *Mycoplasma* spp., *M. felis*, and *M. equirhinis* were found in TW samples from horses, the presence of these pathogens was not associated with clinical signs of IAD. Bond et al. [7] identified 19 different types of bacteria in BALF fluid, and the following phyla accounted for the majority (95.54%) of the bacteria isolated from tracheal specimens: *Proteobacteria* (43.85%), *Firmicutes* (16.82%), *Bacteroidetes* (13.24%), and *Actinobacteria* (21.63%). The relative abundance of *Streptococci* and *Candidatus Saccharibacteria* was higher, whereas the relative abundance of *Psychrobacter*, *Rhodococcus*, *Aerococcus*, and *Hymenobacter* spp. was lower in horses with IAD [7]. Fillion-Bertrand et al. [93] examined the respiratory tract microbiome of healthy and asthmatic horses and found that it was associated with environmental factors. The abundance of *Gammaproteobacteria* was lower in horses under high levels of antigen exposure (*p* = 0.07). Interestingly, the effect of the environment on the lung microbiome was more pronounced in healthy (*p* = 0.004) than in asthmatic horses (*p* = 0.035). According to the authors, the altered lung microbiome in asthma might not be inherent but coincident with inflammation. Xavier et al. [94] noted a significantly higher number of fungi in the respiratory tract of horses affected by RAO than in that of healthy animals.

3. Methods of microbial contamination mitigation

Microbial air contamination depends on the ventilation efficiency in the stable [78,84]. According to Houben [95], natural ventilation is more efficient than mechanical ventilation. Research has also shown that the presence of airborne microorganisms is influenced by the housing system. Microbial contamination was the highest in overpopulated stables and in a box housing system [85]. Bacteria of the genus *Staphylococcus* were the most abundant in horse stables [85] and indoor riding arenas [96], whereas the concentrations of airborne fungi were approximately twice as high in indoor stables than in outdoor paddocks [84]. Reducing microbial counts in the stable environment is an indirect way of reducing the concentrations of gaseous admixtures formed during microbial fermentation—these methods are described above. In addition, it is important to consider the hygienic quality of bedding and feed components, which are discussed in the following sections [80,97]. 

##### Influence of Air Dust

Hot humid air increases bronchial temperature and causes bronchospasms in horses with respiratory inflammations [98,99,100], including asthma [19]. Approximately 30% of the variance in equine asthma prevalence in veterinary hospitals could be explained by the sum of climatic factors and their effect on allergen concentrations in ambient air [61]. In horses, symptoms of asthma may be exacerbated in the presence of organic dust in inhaled air [101].

1. The main sources of aerial particulate matter

The immune response of horses exposed to organic dust from hay and straw has been investigated in recent years [101]. The main causes of excessive dust include insufficient ventilation, as well as activities related to stable management, such as applying bedding material and removing manure or feed [54]. Another source of dust is horsehair and the exfoliated epidermis of horses, especially with improper grooming management [102]. It should be emphasized that horses are exposed to the influence of particulate matter both in stable buildings and in open spaces [9].

2. The impact of dust on the equine respiratory system

Many studies have shown that excessive dust levels in stable air are associated with the occurrence of allergic reactions and respiratory diseases, including EA [23,64]. Aerosol concentrations, their biological and chemical characteristics, and their location in the respiratory system have to be determined to assess horses’ exposure to inhaled particles [103]. Exposure to organic dust elicits a strong reaction in horses with EA, which indicates that the EA-induced impairment of lung function is similar to that noted in asthmatics and farmers chronically exposed to dust. At rest, a horse has a lung capacity of 78 liters of pumped air per minute [101]. Dust particles enter specific structures of the respiratory system, depending on airflow characteristics, which are largely influenced by the geometry of the respiratory tract, the rate and pattern of breathing, and particle characteristics. Large particles (over 10 µm) are usually retained in the upper respiratory tract and are less likely to penetrate the lower respiratory tract. The smallest soluble particles potentially pose the greatest threat to the respiratory system [101,102,103]. Solid particles with a diameter below 10 µm and below 2.5 µm (<PM10 and <PM2.5, respectively) are pro-inflammatory, irritating substances that promote bronchoconstriction [73]. Exposure to respirable dust with a particle size < 2.5 µm, which can reach the bronchi and alveoli when air is inhaled, is the most dangerous for horses. Exposure to increased amounts of PM2.5 is associated with an increase in granulocyte counts in the lower air tract [104]. High levels of PM2.5 in air decrease the efficiency of the respiratory system and undermine horse performance in sports [105]. Not only particle size but also solubility dictate where these pollutants are deposited in the respiratory tract [101,102,103]. Extremely fine aerosols can mediate the phagocytosis-induced apoptosis of macrophages or can avoid phagocytosis and gain access to the blood stream directly through the alveolar wall [90]. The recommended dust levels for horses are based on standards developed for human occupational medicine: 4 mg/m^3^ for all inhalable fractions and 3–10 mg/m^3^ for the alveolar fraction [64,95].

3. Dust mitigation methods

A number of environmental management measures can be taken to limit the exposure of horses to dust. First of all, horses should be provided with access to outdoor runs as much as possible [106,107]. However, it is important to remember that horses are still exposed to dust even when outdoors [9]. It is recommended that indoor activities such as sweeping or bedding be carried out when the animals are outside and that wet cleaning be regularly carried out [108]. In the stable housing system, the use of dust-free bedding, such as wood shavings, peat, or straw pellets, is recommended [81,109,110,111]. Other important factors are the method of feeding and the type of feed [101,112]. It is recommended that hay be soaked [97,106] or steamed [112], which significantly reduces dustiness. Another solution is to replace the hay with high-quality haylage [113]. Pelletized feed and concentrates contain much less dust, which significantly reduces the risk of respiratory irritation [101,114,115]. The activities reducing dust levels in the equine environment are discussed in detail in the subsequent sections.

The influence of air pollutants on the well-being of asthmatic horses is presented in Table 2.

#### 3.3.3. Housing System

It should be noted that housing conditions directly affect the physiological status and, consequently, the well-being of horses [116]. For the above-mentioned reasons, many authors have undertaken research on the level of air dust in stables and the factors influencing this level. These studies demonstrated that the main causes of excessive dust were inadequate ventilation and stable management activities, such as the application of bedding material and the removal of manure or feed [54]. 

Despite the fact that horses kept in stables are more exposed to dust and dirt, Davis and Sheats [9] found that asthma, including mild/moderate asthma, also affects horses that are kept outdoors in pastures. The high prevalence of mild/moderate lower airway inflammation in horses that are kept outdoors has been attributed to continuous access to round bale hay and exposure to air pollution in pastures located in an urban setting and in close proximity to a heavily travelled four-lane road [9]. It has been suggested that asthmatic horses should ideally be kept in pastures all day and fed grass or a completely cubed or pelleted diet [106]. In a study by Simões, free-range asthmatic horses did not present with symptoms such as nasal discharge and cough, did not require pharmacological treatment, and showed a significant improvement in their clinical condition. The time spent outdoors significantly influenced their level of breathing effort, coughing, and medical treatment and led to an overall clinical improvement [106,107]. 

Horses are exposed to dust and dirt regardless of whether they are housed in stables or kept free-range [9]. Sowińska et al. [116] compared the results of blood tests in horses that were kept in stables with and without access to outdoor runs. Based on the analyzed blood indicators, welfare levels were considerably higher in horses that remained outdoors for around 6 h per day. In contrast, the disruption of homeostasis and an increased activation of the immune system were observed in horses that were kept in stables without access to outdoor paddocks. Grzyb et al. [108] compared the concentrations of particulate matter and bacterial aerosols in outdoor runs and box stables and found higher levels of contamination in runs than in box stables. In the cited study, bacterial aerosol concentrations were several times higher inside stables than in outdoor runs. The authors proposed a wet cleaning system to minimize the amount of particulate matter in the stable environment [108].

Horses kept in stables are much more likely to be exposed to high concentrations of airborne irritants than free-range horses [23,117]. The combined effect of feed and bedding on the concentrations of particulate matter and endotoxins was analyzed by comparing horses that were kept on bedding and fed hay with horses that were housed in a low-dust environment on wood shavings and fed pelleted hay [109]. Dust emissions were reduced by over 50% in the latter system. The concentration of selected aeroallergens, such as *Aspergillus fumigatus*, was twice as high in the stables. Similar results were reported by McGorum et al. [117], who analyzed the concentrations of inhaled endotoxins in various housing systems. The concentration of inhaled endotoxins decreased ten-fold when horses were kept in well-ventilated stables on wood shavings and were administered pelleted feed, which reduced dust emissions in the stable environment.

The results reported by Simões highlight the importance of controlling environmental factors when keeping horses with asthma [106]. Despite the above, the owners were reluctant to switch to alternative housing systems that were more beneficial to the respiratory health of horses. Most owners attempted to improve environmental factors by switching to less dusty bedding, which resulted in a significant health improvement in all studied cases. Owners generally find it easier to adopt a single change rather than several changes, and they are particularly reluctant to change their daily habits [106]. In a study by Barton et al. [118], an attempt was made to evaluate the efficacy of glucocorticosteroid treatment combined with dust reduction in the stable environment of the tested horses. It was difficult for the authors to assess the effects because of the difficulties in monitoring the proper care of the horses, which depended primarily on the honesty of the owners in ensuring such rigorous conditions for the welfare of their horses. This confirms that the effective treatment of a horse and its maintenance are the responsibility of the owner himself. 

The influence of housing system and air pollutants on the well-being of asthmatic horses is presented in Table 3.

#### 3.3.4. Different Feeding Systems

The type of feed also affects horses’ exposure to dust [101,112]. Hay, which is the basis of horse nutrition, is a source of fungi and inorganic dust [97,101,107], and it is not recommended for horses kept in a stable [110]. The dust content of hay may vary slightly in subsequent cuttings, but the cutting itself is not a factor that determines dustiness [119]. Fungal contamination in airborne particles and dust contamination are highest in late-cut hay with a high moisture content (rainfall after harvest) or after hay making (baled at 75% DM) [120]. Dry hay potentially poses a greater health risk than silage because it is more likely to be contaminated with mold and *Aspergillus* spp. [121]. *Aspergillus fumigatus*, commonly found in moldy hay, contributes to the exacerbation of RAO symptoms, as does the presence of other fungal species. This indicates that the microbiological quality of hay is an important consideration [122]. Dust emissions can be reduced by 50–60% when hay is immersed in water immediately before feeding [113,123]. However, this procedure is time-consuming and not commonly used in practice [106]. Haylage is an increasingly popular feed material for horses. There is a general scarcity of studies comparing dust exposure in horses fed different forage types [112]. In comparison with dry hay, haylage reduces exposure to respirable dust by 60% to 70% [112,113,120]. Inhalable dust (PM10), as defined by the World Health Organization, refers to particles under 10 μm in diameter. In turn, respirable dust (PM2.5) are particles measuring 2.5 μm or less in size that can be inhaled through the nose or mouth. Respirable dust appears to be more dangerous due to its ability to enter the lower respiratory tract. Olave et al. [112] reported that horses fed haylage had significantly lower BALF neutrophil counts than horses fed dry hay. Soaked or steamed hay reduces respirable dust levels and microbiological pollution [124,125,126]. Simões [106] found that properly soaked hay (for 20–30 min) was associated with a clinical improvement in asthmatic horses. Soaked hay eliminated coughing, clinical signs, and the need for medical treatment [106]. Glatter et al. [97] recommend soaked hay for asthmatic horses directly after treatment. It should be noted, however, that hay soaking is a time-consuming procedure, which could be one of the reasons why it is not commonly used in practice [106]. Moreover, hay steaming reduced respirable dust by 95% and decreased bacterial and mold contents by 99% [97,110,124]. Hay steaming improves feed hygiene, and, in some cases, steamed hay may be the only source of roughage for asthmatic horses [127]. In racing horses, exposure to respirable dust can be reduced by replacing 30% of dry hay with steamed hay or haylage [112]. Larson [128] compared the effects of round bale and cubic bale hay on the equine respiratory system and found that round bale hay increased the risk of respiratory infections. This observation was attributed to higher counts of thermophilic actinomycetes in round bale hay and higher levels of dust exposure. Hay handling and hay composition significantly influence contamination with particulate matter and mold [101]. When properly harvested, white clover (*Trifolium repens*) and winter ryegrass (*Lolium perenne*) have been shown to minimize the release of respirable particulates and mold [120]. In addition, late-cut hay rolled into bales with a dry matter content of 85% and haylage were the least abundant sources of respirable particulates and fungi [120]. In a study by Hunt [114], the concentration of respirable dust particles was reduced by 99% when poor-quality hay was replaced with pelleted feed. Ready-made pellets decreased the concentration of solid particles twelve-fold relative to uncleaned oats, while cleaned oats and steamed barley reduced dust levels by 80% [115]. Crushed barley and oats reduced particle pollution sixty-fold and decreased *Aspergillus fumigatus* counts ten-fold in comparison with ready-made molasses-based concentrates [129]. Concentrated feeds may also contribute to exposure to respirable particulates [101]. Oil-treated hay and alfalfa pelleted hay improve lung function and allow one to control the severity of equine asthma symptoms [130]. The average counts of bacteria, molds, and yeasts are higher in cereals than in compound feeds, and mold counts are significantly higher in grain with a dry matter content below 86% [131]. Research by Nogradi et al. [132] also demonstrated the beneficial effects of omega-3 polyunsaturated fatty acid (PUFA) supplementation on clinical signs, lung function, and airway inflammation in horses with recurrent airway obstruction (RAO) and inflammatory airway disease (IAD). Feeding horses with RAO and IAD a PUFA supplement containing 1.5–3 g docosahexaenoic acid (DHA) for 2 months provides an added benefit to a low-dust diet. Supplementation with DHA resulted in a 65% reduction in the “long” clinical score, which is observed in horses with RAO treated with inhaled corticosteroids and a low-dust diet. 

The influence of nutritional factors on the well-being of asthmatic horses is presented in Table 4.

#### 3.3.5. Bedding Material as a Source of Stable Contaminants

Among many factors that can affect contamination levels, the type of bedding material has also been considered [76,77,95]. Bedding material considerably influences the well-being of horses kept in stables [133]. Stalls are bedded to absorb urine, moisture, and gases and to increase the animals’ welfare [81]. Bedding material should not only ensure hygienic conditions in the stable and exert a positive impact on the stable microclimate, but it should also cater to the behavioral needs of horses, such as digging, picking out plant blades, rolling, or lying down to rest [134,135,136,137]. The price and availability of bedding material, transport costs, and ease of use are also important considerations in the choice of bedding [81,138]. Litter disposal or recycling are equally important considerations, and spent bedding materials can be used as organic fertilizer [139]. Oat, wheat, or barley straw is most commonly used as bedding material in stables [72,134,135,137], even if it is heavily contaminated with fungi and other microbial pollutants [133]. In addition, straw is not as digestible as hay, and in some horses, straw consumption could contribute to gastrointestinal problems such as colic [136]. Straw is one of the main sources of dust in stables [101,107,133]. Wheat straw is the most popular bedding material that contains more dust than oat straw [119]. If improperly stored, straw may also be a source of dangerous molds and their toxins [133]. In the work of Kwiatkowska-Stenzel et al. [133], straw was characterized by having a moderate dustiness and being microbiologically pure, but an endoscopic evaluation of the equine airduct showed changes characteristic of equine asthma. Many horse owners search for more absorbent bedding materials that are easy to clean. Cut straw can be replaced with straw pellets to reduce dust emissions in stables [64]. Straw pellets may also be used to improve the quality of bedding material [72]. However, BALF neutrophil counts were higher in horses kept on straw pellet bedding than on peat, which may increase the risk of asthma symptoms. Wood shavings offer a promising alternative to straw [81,111,134,140,141]. However, wood shavings do not compost easily and could be more difficult to dispose than straw [142]. Wood shavings are obtained by processing coniferous trees. They are additionally dedusted and are free from mold and fungi. In practice, wood shavings are quite easy to keep clean, but solid excreta must be removed daily. In a study by Kwiatkowska-Stenzel et al. [133], horses kept on wood shavings had the most desirable endoscopic outcomes. The above finding could be attributed to the low dustiness of this bedding material [110]. Peat is also a good source of bedding for horses with respiratory problems. Peat has a naturally low pH, which inhibits microbial growth in litter [81]. According to Saastamoinen et al. [81], peat is superior to wood shavings because it binds to ammonia, decreasing the ammonia concentration in stables. Peat has a low pH level, which inhibits microbial growth in litter. BALF neutrophil counts were higher in horses kept on loosely stored peat than on baled peat, which may increase their risk of asthma symptoms. Peat is superior to straw pellets in terms of respiratory health [111,143]. It is dedusted, free from dust and impurities, and recommended for horses with respiratory diseases. Peat is very easy to make, and it is easy to clean and remove due to its dryness and flowability. Some peat mulches also contain essential oils (such as pine oil), which not only have a positive effect on the respiratory tract, but also inhibit the development of fungi and molds in the substrate, which reduces the amount of allergens in the environment [119]. Despite their high absorption capacity, paper products are less commonly used as bedding. One study found that paper absorbed three times more water than wood shavings [144]. 

The influence of bedding material on the well-being of asthmatic horses is presented in Table 5. 

## 4. Summary and Conclusions

Asthma is a common respiratory disease of humans and animals, including horses. Both mild and severe asthma undermines the physical performance of horses and causes financial losses for owners. The symptoms of equine asthma are typical of respiratory inflammations, and they include coughing, nasal discharge, and breathing difficulty even at rest in horses with severe asthma. BALF and tracheal wash analyses are the most popular methods of diagnosing equine asthma. Inadequate housing conditions undoubtedly increase the risk of asthma. Air pollutants of various origin not only increase the risk of the disease, but also exacerbate its symptoms. Equine asthma is strongly linked with the well-being of horses, which is compromised in affected animals. For this reason, horse owners, farm employees, and the equestrian industry should be educated about proper living and training conditions for horses. The well-being and physical performance of horses can be significantly improved by changing daily habits in horse maintenance, and these changes can reduce costs for horse owners, as well as improve the health of these sensitive animals.

## 5. Directions for Future Research

Equine asthma is a significant health problem for horses and horse owners. Despite the fact that considerable research has been conducted to identify the factors that cause and exacerbate equine asthma, owners are reluctant to introduce permanent changes in daily horse maintenance to improve the well-being of horses. In some cases, such changes could increase cost of maintenance or labor inputs in daily grooming. However, numerous research studies have shown that environmental conditions have the greatest impact on the health of asthmatic horses. Further research is needed to identify potential causative factors of equine asthma, and educational campaigns should be implemented to increase public awareness of the problem. A healthy and comfortable environment will improve the well-being of horses, thus also maximizing the benefits of keeping them. Furthermore, equine asthma is an important topic of research because horses can be used as an animal model for human asthma.

Pollution, in particular in stables, is one of the key determinants of equine health that contributes to asthma, decreasing the well-being of horses and their use by humans. Therefore, effective methods of reducing pollution should be developed, and the existing solutions should be improved. This research topic should be prioritized in the future, and it will be continued by the authors.

## Figures and Tables

**Table 1:** The influence of climatic factors on the well-being of asthmatic horses.

Potential Factor	Conclusion	Equine Asthma Subtype of Horses Participating in the Research to Which the Conclusions Refer	References
Temperature and humidity	Low temperatures help reduce the severity of asthma symptoms	SEA	[19]
	Exercising horses in cold air has a positive effect on their respiratory health	Clinically healthy	[58]
Season	Asthma symptoms may become more severe in the winter, depending on additional factors related to how the horses are kept	SEA + MEA	[9]
	BALF cytology results vary across seasons	MEA	[57]
	Keeping horses stabled in winter contributes to changes in BALF cytology results and mucus secretion	Clinically healthy	[59]

**Table 2:** The influence of air pollutants on the well-being of asthmatic horses.

Potential Factor	Conclusion	Equine Asthma Subtype of Horses Participating in the Research/to Which the Conclusions Refer	References
Gases	Selected harmful gases commonly found in stable air negatively affect the health of horses. The highest concentrations of naturally occurring gaseous compounds were noted in early morning (4.00 a.m.)	Not mentioned	[65,70,71,72,73]
Microorganisms	Airborne microorganisms, including *Penicillium* spp. and *Fusarium* spp., and G- bacteria produce toxins that are dangerous to animals’ health, including their respiratory health	Not mentioned	[81,82]
Dusts	Keeping horses in winter involves feeding them hay and bedding, which increases their exposure to dust	SEA	[60]
	Horses’ athletic performance is correlated with pollutants in the air they breathe	Not mentioned	[62]
PM10	Large particles of organic dust from hay and straw are usually retained in the upper respiratory tract and are less likely to penetrate the lower respiratory tract	MEA + SEA	[100]
PM2.5	The presence of PM2.5 particles affects BALF cytology results	MEA	[103]
	Fine dust particles reduce the efficiency of the respiratory system and consequently worsen horses’ performance in sports	MEA	[104]

**Table 3:** The influence of housing system on the well-being of asthmatic horses.

Potential Factor	Conclusion	Equine Asthma Subtype of Horses Participating in the Research to Which the Conclusions Refer	References
Housing	Pastures located next to busy streets expose horses to high concentrations of dust in the air they inhale.	Not mentioned	[9]
	The concentrations of airborne microorganisms vary across housing systems.	Clinically healthy	[85]
	Asthmatic horses should be kept outdoors and freely graze year round.	MEA + SEA	[119]
	Access to pasture improves horse welfare.	Clinically healthy	[116]
	Changes in daily housing habits can significantly improve respiratory health in horses.	SEA	[106]
	Keeping horses in outdoor paddocks has a positive impact on the health of their respiratory system.	Not mentioned	[107]
	Bacterial aerosol concentrations are several times higher inside stables than in outdoor runs.	Not mentioned	[108]
	Stable breeding increases the exposure of horses to airborne irritants.	Not mentioned	[117]

**Table 4:** The influence of nutritional factors on the well-being of asthmatic horses.

Potential Factor	Conclusion	Equine Asthma Subtype of Horses Participating in the Research to Which the Conclusions Refer	References
Dry hay	Dry hay is not recommended for stabled horses.	Not mentioned	[110]
	Native hay has high counts of typical bacterial, fungal, and yeast species.	Not mentioned/Clinically healthy	[97,107]
	Dry hay containing mold and *Aspergillus* spp. is potentially more dangerous to horse health than silage.	Not mentioned	[121]
	Feeding horses dry hay causes changes in BALF cytology results.	MEA	[112]
	Feeding horses baled hay triggers an increased respiratory defense response, compared with hay cubes of the same quality.	Clinically healthy	[128]
	Dust content differs in hay from subsequent cuttings.	MEA + SEA	[119]
	The timing of grass mowing affects the levels of fungal and dust contamination in the hay.	SEA	[120]
Oil-treated hay and alfalfa pelleted hay	The addition of soybean oil has a positive effect on the equine respiratory system and reduces the severity of asthma symptoms.	SEA	[130]
Steamed hay	Steaming hay improves feed hygiene.	MEA + SEA	[127]
	Hay steaming reduces up to 95% of respirable dust in the horse’s breathing zone during consumption compared with dry hay.	SEA	[110,124]
	Hay steaming effectively reduced and stabilized yeast and mold counts during storage, unlike dry hay.	SEA	[97,124]
Soaked hay	Soaked hay should be administered directly after treatment due to increasing levels of microbiological contamination.	Clinically healthy	[97]
Haylage	Haylage is least likely to produce airborne particles compared with dry hay.	SEA	[120]
Concentrates	Oats may be contaminated with molds which provoke coughing in horses.	Not mentioned	[131]
PUFA	Omega-3 polyunsaturated fatty acid (PUFA) supplementation alleviates the symptoms of respiratory disorders.	MEA + SEA	[132]

**Table 5:** The influence of bedding material on the well-being of asthmatic horses.

Potential Factor	Conclusion	Equine Asthma Subtype of Horses Participating in the Research to Which the Conclusions Refer	References
Straw (native)	Straw stored under inadequate conditions may be a source of microbial contamination.	Clinically healthy	[133]
	Wheat straw is characterized by higher dusting than oat straw.	MEA + SEA	[119]
	Straw contains significantly more dust, endotoxins, and fungi than wood shavings.	Clinically healthy	[107]
Straw pellets	Horses kept on straw pellets show higher numbers of neutrophils in BALF cytology than those kept on peat.	Clinically healthy	[111]
Wood shavings	Wood shavings decrease the content of dust in stable air.	Not mentioned	[110]
Peat	Peat is less likely to cause lower airway neutrophilia in horses.	Clinically healthy	[143]
	Peat stored loosely produces more dust than when pressed, which is confirmed by BALF cytology results.	Clinically healthy	[111]
	The addition of selected essential oils to peat facilitates breathing in horses and limits the development of microorganisms in the bedding.	MEA + SEA	[119]
	Peat reduces ammonia concentration in the stable environment and further improves the well-being of horses.	MEA + SEA	[81]
